# Separated at Birth: The Politics of Pharmacare for All in Canada and Medicare for All in the United States Comment on "Universal Pharmacare in Canada"

**DOI:** 10.34172/ijhpm.2020.31

**Published:** 2020-03-07

**Authors:** Carolyn Hughes Tuohy

**Affiliations:** Munk School of Global Affairs and Public Policy, University of Toronto, Toronto, ON, Canada.

**Keywords:** Healthcare Politics, Universal Pharmacare, Medicare for All, Canada, United States

## Abstract

Policy decisions about healthcare coverage in Canada and the United States in the 1960s placed two virtually identical systems on different evolutionary paths in the physician and hospital sectors. However, prescription drug coverage remained outside Canada’s single-payer model, and employer-based coverage continued to be the norm for the workforce population, as is the case across the broad healthcare system in the United States. As a result the current debate about pharmacare in Canada mirrors in political microcosm the larger debate on universal health insurance among American Democrats. In each case the near-term prospects for a single-payer plan appear slim.

The current debate about pharmacare in Canada, as represented in recent articles in this journal by Hajizadeh and Edmonds^1^ and by Lewis,^2^ mirrors in political microcosm in the drug sector the larger debate on universal health insurance across the broader healthcare arena among American Democrats. Those who argue for a federal comprehensive single-payer pharmacare plan in Canada have their counterparts in the advocates of “Medicare for All” in the United States. Conversely, those who would instead achieve universal coverage for drugs through a multi-payer combination of public and regulated private coverage in Canada share a somewhat similar mindset with the advocates of building on the current “Obamacare” model in the United States.

These parallels can be traced back to policy decisions taken in the 1950s and 1960s in both countries. At the time, the Canadian and American healthcare systems were as similar as those of any two countries on the planet.^3^ Not only were the delivery systems identically based on private fee-for-service medical practice and voluntary and public hospitals (accredited by the same bodies), but the systems of healthcare finance also showed roughly similar levels of private insurance coverage, largely employer-based, and means-tested public coverage. Both countries adopted single-payer public programs of physician and hospital insurance: that is, systems characterized by public payment of private providers. But for reasons grounded in the macro-politics of the time, Canada’s single-payer program extended to the entire population, while American Medicare and Medicaid were limited to the elderly and social assistance recipients respectively. This “parting at the crossroads”^4^ took the Canadian and American physician and hospital sectors in very different directions. However, the Canadian prescription drug sector, including its politics, remained outside the single-payer system.

In the following decades, the Canadian single-payer system for physician and hospital coverage became entrenched, drawing the medical profession and the state into a tight negotiating arrangement in each province, and gaining broad and deep support in public opinion. Outside the single-payer system, however, the bulk of the workforce population continued to rely on private employer-based coverage for prescription drugs, while various public programs of drug coverage for the elderly and social assistance recipients were adopted at the provincial level. Meanwhile, in the United States, private employer-based coverage continued to be the norm for the workforce population across the entire health system.

The result was that, in terms of coverage, the Canadian drug sector looked a lot like the American system as a whole. The parallels were not exact: some provinces shifted from age-based to income-scaled coverage for drugs in the 2000s, and public drug coverage for elderly Americans was not widespread Part D in 2003 and its enhancement under the Affordable Care Act in 2010. But the fundamental commonality – the entrenchment of employer-based private coverage as the norm for the workforce population – remained the same in the Canadian drug sector and the broader US system. By the early 2000s, an estimated 60% of all Canadian workers and their families and 26% of retirees over age 65 had employer-sponsored coverage for drugs,^5^ matching almost exactly the pattern for the healthcare sector as a whole in the United States, where 62% of non-elderly Americans had employer-based health insurance in 2004.^6^ By 2018, private insurance accounted for 37% of total spending on prescription drugs in Canada,^7^ making the Canadian drug sector very similar to the US healthcare arena as a whole, where 34% of total health spending (and 40% of prescription drug spending) was accounted for by private insurance in 2018.^8^ Private out-of-pocket spending made up another 21% of total drug expenditure in Canada and 10% of overall health expenditure (14% in the case of drugs) in the United States.

Meanwhile, the quest for universal single-payer coverage – across-the-board in the United States and extended to prescription drugs in Canada – has never been abandoned and periodically peaks in each country. The most recent peaks have occurred almost simultaneously in both countries, with the recommendation for a single-payer plan of drug coverage from the federal Advisory Council on the Implementation of National Pharmacare (the Hoskins Commission)^9^ in Canada, and the championing of “Medicare for All” by Democratic presidential candidates Bernie Sanders and Elizabeth Warren in the United States.

Public support for such proposals, however, has historically been broad but shallow. In 2002, for example, in a survey conducted in the context of a federal commission of inquiry, “creating a new national pharmacare program to help people pay for their prescription drugs” ranked fifth in a list of seven potential priorities for more public spending on healthcare. Only 33 percent saw it as a top priority, as compared with 63% citing reducing waiting times for diagnostic imaging. In 2004, universal drug coverage ranked 12th of 13 potential priorities to “improve the quality of care.”^10^ In a 2015 poll asking only about support for universal drug coverage without offering any competing priorities found overwhelming (87%) support for “adding prescription drugs to Medicare” (that is, extending the single-payer model). But majorities also opposed funding this expansion through premiums or increased sales or personal income taxes.^11^ US polling has also consistently found support for a single-payer plan to be highly vulnerable to the presenting of supporting or opposing arguments. In a 2015 poll, support for a single-payer plan dropped from 55% to 40% once the prospect that “many Americans would pay more in taxes” was raised.^12^ Similarly, a 2017 poll by the Kaiser Family Foundation showed that describing potential benefits or costs of a single-player plan could respectively raise support to 71% or opposition to 62 percent.^13^ Subsequent polling found continuing malleability and misunderstanding.^14^ The degree to which public opinion actually shapes public policy is very much a matter of debate among political scientists.^15,16^ Nonetheless, the vulnerability of public opinion to alternative framings of single-payer plans in both Canada and the United States suggests that at the very least advocates face significant challenges of shoring up support in the face of political opposition.

The prospects for Medicare-for-all in the United States appear slim. Even its most ardent advocates such as Senators Sanders and Warren have tempered their proposals as noted below. The politics of US healthcare require building coalitions of interests and individual legislators with deep independent bases of political support. Instituting a comprehensive single-payer plan in the United States would therefore likely require that it be part of a “sea-change” reform agenda in American politics akin to the Progressive era or the New Deal – a wave of change powerful enough to wash over those independent power bases. As James Morone has put it, Medicare for All “is more than a health policy prescription. … It is a policy proposal designed to improve healthcare delivery, an ambitious claim about equality and social justice, and an effort to usher in a more progressive era in American politics. Each is a long shot, but Medicare for All and its advocates stand in a venerable reform tradition that has rewritten US politics many times in the past.”^17^

The politics of pharmacare in Canada are not identical to those of the broad US arena (for one thing, opinion on healthcare generally is much less polarized by partisanship in Canada than in the United States^18^); but still daunting. In Canada the independent veto-wielding government players are federal and provincial governments, not individual legislators. Single-payer physician services insurance (which marked the true birth of Canadian medicare) was adopted in 1966 in rare political circumstances. At the federal level, a strong Liberal minority government was supported by the progressive New Democratic Party. The federal Liberal party itself was seeking to rebuild around a progressive social policy agenda after its discredited “business” wing had taken the party to successive electoral defeats. Even more important, however, was the federal-provincial climate: although hardly without conflict, this was in historical perspective the golden era of “cooperative federalism” in which agendas of “province-building” rendered provincial governments eager for federal transfers and open to negotiating the terms.

Today, the federal government’s position in a strong minority supported by the New Democratic Party mirrors the circumstances of the 1960s. But federal-provincial politics are very different now, and healthcare is not at the top of provincial agendas dominated by climate change policies, pipelines and federal revenue equalization transfers. At their annual meeting in November 2019, the premiers could come to no consensus on pharmacare and could agree only on seeking an increase in the annual rate at which federal health transfers are increased. Appeals to equity and social justice such as those issued by Hajizadeh and Edmonds, and especially by Lewis are compelling, but they imply the sort of “sea change” that can create the tide on which the politics of building a broad federal-provincial coalition can ride. In the absence of such a change, the route charted by the Hoskins Commission appears tortuous. Navigating that route may need to await a new government and a new mandate.

Absent a “sea change,” a more incremental step – such as the establishment of a public plan available as an alternative to private insurance – might hold more promise as a route to universal coverage. Proponents of such a “public option” proposal in the United States failed to gain its inclusion under the Affordable Care Act in the heated debates of 2009-2010, largely because it was admittedly seen as a precursor to a full single-payer plan.^19^ Now, however, the proposal has been resurrected by several candidates for the Democratic presidential nomination, and even Sanders and Warren would establish a “public option” as a transitional step to a full single-payer plan. In Canada, the province of Quebec’s framework of mandatory universal coverage for drugs includes a public plan for those without employer-based insurance, as discussed in several other contributions to this series, and could provide a template for diffusion across provinces through a federal framework.

No cross-national comparison is exact. Nonetheless, the remarkable similarities between the politics of pharmacare for all in Canada and Medicare for all in the United States suggest a degree of caution in each case. Each proposal would mean the extension of the single-payer programs initially established in the 1960s and now knit into the fabric of public and private coverage – in the Canadian case the extension of the universal single-payer plan to include prescription drugs, and in the American case the extension of a plan for the elderly and disabled to the full population. For Canadians, witnessing the current debate in the United States underlines the political challenges faced by those who would replace well-established private coverage with a public plan. For Americans, the fact that even Canada, the birthplace and exemplar of single-payer coverage, has been politically unable to extend that model provides a cautionary tale.

## Ethical issues

Not applicable.

## Competing interests

Author declares that she has no competing interests.

## Author’s contribution

CHT is the single author of the paper.
